# Health care costs in the elderly in Germany: an analysis applying Andersen’s behavioral model of health care utilization

**DOI:** 10.1186/1472-6963-14-71

**Published:** 2014-02-14

**Authors:** Dirk Heider, Herbert Matschinger, Heiko Müller, Kai-Uwe Saum, Renate Quinzler, Walter Emil Haefeli, Beate Wild, Thomas Lehnert, Hermann Brenner, Hans-Helmut König

**Affiliations:** 1 Department of Health Economics and Health Services Research, Hamburg Center for Health Economics, University Medical Center Hamburg-Eppendorf, Martinistr 52, Hamburg, 20246, Germany; 2 Department of Social Medicine, Occupational Medicine and Public Health, University of Leipzig, Leipzig, Germany; 3 Divisions of Clinical Epidemiology and Aging Research and Preventive Oncology, German Cancer Research Center, Heidelberg, Germany; 4 Department of Clinical Pharmacology and Pharmacoepidemiology, University of Heidelberg, Heidelberg, Germany; 5 Department of General Internal Medicine and Psychosomatics, Medical University Hospital Heidelberg, Heidelberg, Germany

**Keywords:** Cost of illness study, Cross-sectional study, Health care utilization, Health care costs, Multimorbidity, Elderly, Andersen behavioral model

## Abstract

**Background:**

To analyze the association of health care costs with predisposing, enabling, and need factors, as defined by Andersen’s behavioral model of health care utilization, in the German elderly population.

**Methods:**

Using a cross-sectional design, cost data of 3,124 participants aged 57–84 years in the 8-year-follow-up of the ESTHER cohort study were analyzed. Health care utilization in a 3-month period was assessed retrospectively through an interview conducted by trained study physicians at respondents’ homes. Unit costs were applied to calculate health care costs from the societal perspective. Socio-demographic and health-related variables were categorized as predisposing, enabling, or need factors as defined by the Andersen model. Multimorbidity was measured by the Cumulative Illness Rating Scale for Geriatrics (CIRS-G). Mental health status was measured by the SF-12 mental component summary (MCS) score. Sector-specific costs were analyzed by means of multiple Tobit regression models.

**Results:**

Mean total costs per respondent were 889 € for the 3-month period. The CIRS-G score and the SF-12 MCS score representing the need factor in the Andersen model were consistently associated with total, inpatient, outpatient and nursing costs. Among the predisposing factors, age was positively associated with outpatient costs, nursing costs, and total costs, and the BMI was associated with outpatient costs.

**Conclusions:**

Multimorbidity and mental health status, both reflecting the need factor in the Andersen model, were the dominant predictors of health care costs. Predisposing and enabling factors had comparatively little impact on health care costs, possibly due to the characteristics of the German social health insurance system. Overall, the variables used in the Andersen model explained only little of the total variance in health care costs.

## Background

Due to demographic change, the proportion of elderly people in developed countries will increase substantially in the next decades [1]. Germany is one of the countries most strongly affected by demographic change with the proportion of people aged ≥ 65 years expected to rise by about 50% until 2030 [2]. Due to the progressive increase in the proportion of elderly people, health care systems are faced with serious organizational and financial challenges [3-5]. For a better understanding of the future demand for health care services and health care costs, it is necessary to understand the specific mechanisms that determine the utilization of health care in the elderly.

As health care utilization is influenced by multiple individual and contextual factors, a reasonable starting point for analyzing health care utilization and costs is to define a theoretical framework. There are several explanatory frameworks identifying predictors of health care utilization [6]. One of the most comprehensive and widely used frameworks is the behavioral model developed by R. Andersen and J.F. Newman in 1973 [7]. Therein the authors present a causal ordering of health care utilization within an integrated framework. In the model which has been discussed and continuously refined over the years [8-10], it is assumed that individuals’ use of services is a function of their predisposition to use services (predisposing factors), factors that support or impede use (enabling factors), as well as their need for health care (illness level). Predisposing variables pertain to socio-demographic (e.g. age, sex, education, marital status) and belief characteristics (e.g. values concerning health and illnesses measurable in consequence such as smoking behavior, alcohol consumption, or body mass index) while enabling factors are those that support or impede health care service use (e.g. income, type of health insurance).

According to Andersen and Newman [7], patients’ illness level (representing the need factor) is considered the major determinant of health care utilization. In the elderly, the illness level is often shaped by multimorbidity (MM), defined as the co-occurrence of two or more chronic conditions in one person without reference to an index disease [11]. Among the population aged 65+ the prevalence of MM has been reported to exceed 65% [12-16]. Approaches to measure MM have quantified the number of affected clinically relevant physiological systems weighted by severity [17]. Thus, in the context of the Andersen model, patients’ illness level may be described by a measure of MM rather than individual conditions. Linking MM with health care service use can be a useful strategy for health services research in general populations where the focus is on care and costs of the patient as a whole rather than on the treatment of particular diseases [18,19].

A recent review of the literature asserted the positive association between MM and health care utilization or costs in the elderly and pointed out that in studies conducting multivariate analyses MM typically had a much stronger impact on health care utilization than variables operationalizing predisposing and enabling factors [20]. However, in many of these analyses, predisposing and enabling factors such as age, gender, living arrangement, and health insurance status were still significantly associated with health service use independent of MM [20]. Another recent review of studies that specifically applied the Andersen model to analyse health service use in various populations pointed out inconsistencies in the strength and direction of associations which seemed to be strongly influenced by the study context and the characteristics of the study population [21]. Yet, of 16 studies included in this review, none focussed on the elderly population and only one was conducted in Germany [22].

The purpose of our study was therefore to analyze the association of health care costs with predisposing, enabling and need factors as defined by Andersen’s behavioral model of health care utilization in a random sample of the elderly population in Germany. As the German health care system aims at providing universal access to comprehensive health care services, we were interested in the relative impact of predisposing, enabling, and need factors in Germany as compared to findings from the international literature. We hypothesize that the need factor is much stronger associated with health care costs in the elderly in Germany than predisposing and enabling factors.

## Methods

### Sample

This cross-sectional analysis was performed based on 8-year follow-up data from the ESTHER study, a large population-based prospective cohort study conducted in the German federal state of Saarland. The ethics committees of the Medical Faculty, University of Heidelberg, and of the Medical Association of Saarland approved the study which was conducted in accordance with the declaration of Helsinki. Written informed consent was obtained from each participant.

Detailed information about the study design and the participants of ESTHER has been reported elsewhere [23,24]. Briefly, baseline recruitment was conducted between July 2000 and December 2002 and included 9,949 participants aged 50 to 74 years. Standardized questionnaires on socio-demographic, medical, and lifestyle factors provided in a postal survey were completed at the baseline assessment and three follow-ups (2, 5, and 8 years). Until the 8-year follow-up 499 individuals deceased and 680 withdrew informed consent, 505 of them due to health reasons.

6,063 individuals participated in the 8-year follow-up (73.4% response rate) completing the standardized questionnaires. For 5,056 of these participants, additional information was collected with a questionnaire provided to their GPs. In addition, all 6,063 participants were asked to take part in an 8-hour geriatric assessment conducted at their homes by trained study physicians, which also included the assessment of health service use to be used for the present analysis (see below). 3,124 individuals participated in this geriatric assessment between July 2008 and December 2010. The analysis presented here is based on these 3,124 individuals.

### Variables

Adequate operationalization and selection of variables representing the Andersen model was ensured by considering Andersen’s own suggestions [8] as well as the results of literature reviews by de Boer [25] and Babitsch [21], which were both largely based on the framework of Andersen’s behavioral model.

#### Predisposing factors

Socio-demographic data include marital status and education (years of schooling) supposed to measure social structure. Age, gender and body mass index (BMI) represent biological factors that determine a person’s likelihood of health care utilization. It was calculated based on participants’ height and weight (kg/m^2^) as measured by the study physician during the geriatric assessment.

#### Enabling factors

Characterized by Andersen as a personal enabling factor, the participants’ income is reported as the square root equivalence scale (SRES), which divides the household income by the square root of the household size [26]. Since the income was assessed per month, the amount of the SRES was multiplied by 3 to obtain the income for a 3-month period (see below).

Another personal enabling factor is the participants’ health insurance. In Germany health insurance is mandatory for the entire population. Approximately 88% of the German population – in particular employees below a certain income ceiling and their family members – are insured by the statutory health insurance (SHI) and keep this insurance after retiring. Self-employed persons and employees above the income ceiling can opt for private health insurance (PHI) and usually stay privately insured after retiring. Regardless of the type of health insurance, all beneficiaries have access to outpatient physician and non-physician services, hospital care, rehabilitation, dental care, prescription drugs, medical supplies and long-term nursing care. For those insured by the SHI there are co-payments for most services but cost-sharing is limited to 2 per cent of household income per year or even 1 per cent for the chronically ill. Accordingly, health insurance was divided into the categories 'statutory’ and 'private’.

To assess the degree of social isolation and thereby a community enabling factor, the LSNS-6 [27], a 6-item short form of the Lubben Social Network Scale [28] was administered to the respondents. The LSNS was specifically developed for older adult populations. Its single item categories are defined as “none”, ”one”, “two”, “three or four”, “five to eight”, “nine or more” persons from family or friends to whom respondents had contact during a one month period. Equally weighting the sum of the six items, the LSNS-6’s total score ranges from 0–30 while the subscale for family and friends ranges from 0–15.

#### Need

To assess illness level as an indicator of participants’ objective need of health service use, the Cumulative Illness Rating Scale for Geriatrics (CIRS-G) [29] was used. The CIRS-G is a modified version of the Cumulative Illness Rating Scale [30], which is a well-established measure of MM in the elderly. On the CIRS-G, 13 categories referring to clinically relevant physiological systems and 1 category referring to psychiatric illness are rated on a five-point severity scale ranging from 0 (no problem) to 4 (extremely severe). The 13 physiological systems are 1. heart, 2. vascular, 3. hematopoetic, 4. respiratory, 5. eyes, ears, nose, throat and larynx, 6. upper gastrointestinal tract, 7. lower gastrointestinal tract, 8. liver, 9. renal, 10. genitourinary, 11. musculoskeletal/integument, 12. neurological, 13. endocrine/metabolic and breast. Assuming that the impact of the categories is additive, a total score from the sum of each of the 14 single categories can be constructed. This total score theoretically ranges from 0 to 56 and simultaneously accounts for the number of diseases and their severity [17]. The CIRS-G questionnaire was completed by respondents’ GPs.

Furthermore, since mental health is only reflected to a small extent in the CIRS-G, subjective mental health status was measured based on the SF-12-questionnaire, a widely used generic questionnaire that does not focus on specific disease groups. The SF-12 is a downsized version of the 36 short form health survey (SF-36), in which a subset of 12 items/questions (of the original 36 contained within the SF-36) is used to derive one summary score each for physical health (PCS score) and for mental health (MCS score) [31]. By covering the same dimensions as the SF-36, i.e. physical functioning (2 questions), role-physical functioning (2 questions), bodily pain (1 question), general health (1 question), vitality (1 question), social functioning (1 question), role-emotional functioning (2 questions), and mental health (2 questions), while using only one-third of the items, the SF-12 is able to produce the two summary scores originally developed for the SF-36 with remarkable accuracy but far less respondent burden [32]. The SF-12 allows to calculate a mental component summary score (MCS) for mental health. The score ranges from 0 to 100 and is standardized to population norms (based on a US norm-sample), with the mean score set at 50 (SD = 10); lower scores indicate worse, and higher scores better mental health. The SF-12 has good psychometric properties [31] and measures subjective Health-Related Quality of Life (HRQOL).

### Health care use

Based on a questionnaire on health service utilization developed by our working group on the specific demands of the German health care system and used in previous studies [33,34], a short health economic questionnaire, especially suited for the application in large epidemiologic studies, was developed within the project. The questionnaire is available from the authors upon request. It covers in-patient care, out-patient physician services, out-patient non-physician services (physical or occupational therapy), medical supplies and dental prostheses, formal and informal nursing care as well as out-of-pocket expenses for the corresponding categories (Table 1). Assessment was retrospective for a period of 3 months for all resources and services. In order to minimize recall bias, the questionnaire contains lists of common health services and goods used in old age. Pharmaceuticals were recorded during home visits by the study doctors by means of a barcode reader when respondents had packages available and by hand otherwise. Missing drug codes were searched using available information on trade name and pharmaceutical form. The health economic questionnaire was administered in personal interviews conducted by the study physicians during the geriatric assessment at respondents’ homes.

**Table 1:** Recorded resources, units of measurement and unit costs used for calculation of costs in the primary analysis

**Sector**	**Resources**	**Units**	**Unit costs (source)**
In-patient treatment	Stays in general hospitals, specialized psychiatric and neurological hospitals or rehabilitation clinics (including day patient treatment)	Days in hospital	Calculated costs of care per day by type (Federal Statistical Office, German Hospital Federation, Statutory Pension Insurance Fund) [35-37]
Out-patient physician treatment	Treatment by GPs, specialists and out-patient clinics	Number of contacts	Calculated costs per contact, by specialization [38]
Other out-patient treatment	E.g. physiotherapy, massage, occupational therapy, non-medical practitioner	Number of contacts	Reimbursement schedules (Statutory health insurance funds; [39-41], calculated costs per contact [38], by type, schedule of fees (Federal office of administration) [42]
Medical supplies and dental prostheses	E.g. walkers, incontinence pads, hearing aids, surgical stockings, dental bridge, crown	Quantity	Reimbursement schedules (Statutory health insurance funds, Federal Association of Panel Dentists; [43,44], calculated costs per item [38], by type
Pharmaceuticals	Specific products (including trade name, drug code, package size, pharmaceutical form, dosage)	Quantity	Pharmacy retail prices (MMI-Pharmindex, Medizinverlag Medizinische Medien Informations GmbH (MMI, Neu-Isenburg) [45]
Nursing home care	Nursing home stays (residential and day care)	Days	Calculated costs of care per day (Federal Statistical Office) [46], by type
Professional home care	Care and assistance provided by professional nursing services and other paid help, differentiated by type (e.g. basic care, assistance with cleaning, shopping, financial matters etc.) and limited to care or assistance required owing to illness or age	Hours	Hourly gross wage rate plus non-wage labor costs for employees in the domain of care and assistance for the elderly or handicapped (Federal Statistical Office) [47,48]
Informal care	Care and assistance provided by family or friends, differentiated by type and limited to care or assistance required owing to illness or age	Hours	Replacement cost method: Hourly gross wage rate plus non-wage labor costs for employees in the domain of care and assistance for the elderly or handicapped (Federal Statistical Office) [47,48]

### Health care costs

By recording all used resources and services, regardless of whether they were covered by health or nursing care insurance or paid for out-of-pocket, a societal perspective was adopted in this analysis. The cost categories analyzed in this study are direct costs of illness arising from the use of resources. Costs were calculated from resource use as recorded in the questionnaire by means of unit costs. Resource categories and sources of unit costs are listed in Table 1.

Pharmaceuticals were monetarily valued using German PZN-codes as recorded in the home visit in conjunction with the MMI Pharmindex database [45]. Medication taken occasionally was valued by means of the pharmacy retail price of one package per 3 months (using the package size as recorded). For continuous medication, costs per unit of the drug were derived from the recorded package size and the corresponding pharmacy retail price, and 3-month costs were obtained by multiplication of the unit cost with the total dose for the 3-month period.

Informal care was valued using the replacement cost approach, i.e. it was assumed that the same amount of care by professional nursing services would have had to be paid for in the absence of an informal caregiver. Accordingly, hours of informal care were valued using the same hourly wage rate as for professional home care. Methods for the valuation of informal care are discussed by van den Berg et al. [49].

Costs were calculated in € at 2009 price levels. Unit costs that were unavailable at year 2009 values were inflated or deflated to year 2009 price levels by means of the consumer price index [50].

For statistical analysis, we categorized cost data as follows: 1) Costs of inpatient care comprising inpatient treatment in general hospitals, specialized psychiatric and neurological hospitals or rehabilitation hospitals; 2) costs of outpatient care comprising outpatient physician treatment, other outpatient treatment, medical supplies and dental prostheses; 3) costs of medication comprising pharmaceuticals; 4) costs of nursing care comprising nursing home care, professional community nursing care and informal care.

### Missing values

With the exception of items of the health economic questionnaire, missing values were imputed by means of multiple imputation by chained equations using the program ice [51] in STATA Release 12 [52]. The last column in Table 2 gives an overview of the percentages of missing values in the single variables. For the imputation procedure a cycle length of 200 was chosen. A graphical analysis showed that this was sufficient for convergence of the imputations. Following the suggestions of van Buuren [53,54], we calculated 100 imputation steps as the recommendation of only 5 imputations by Rubin [55] seemed to be too small for the task. Beyond the variables contained in Table 2, the following variables were entered into the multiple imputation model: SF-12 physical component score, Barthel Index, Mini Mental State Examination score, smoking status, alcohol intake and hand strength. The single sectors of health care costs were part of the imputation model but were not imputed themselves. For the imputation the level of measurement of the particular variables was considered. Imputed values were restricted to the theoretical range of the original variable.

**Table 2:** Socio-demographic and health related sample characteristics (grouped by predisposing characteristics, enabling resources and need)

**Characteristics**		**All**	**Male**	**Female**	**p-value**^**a**^	**Missing values (%)**
		**n = 3,124**	**n = 1,481**	**n = 1,643**		
Predisposing characteristics	Age					
	Mean (SD)	69.6 (6.3)	70.0 (6.2)	69.3 (6.4)	<0.01	0.0
	Range	57-84	58-83	57-84		
	Marital status: n (%)					
	Single	105 (3.4)	54 (3.7)	51 (3.1)	<0.01	1.2
	Married	2,217 (71.8)	1,244 (85.0)	973 (59.9)		
	Divorced	229 (7.4)	66 (4.5)	163 (10.0)		
	Widowed	536 (17.4)	99 (6.8)	437 (26.9)		
	Education: n (%)					
	≤ 9 years of schooling	2,038 (66.2)	916 (62.7)	1,122 (69.3)	<0.01	1.4
	10-11 years of schooling	550 (17.9)	211 (14.5)	339 (20.9)		
	≥ 12 years of schooling	491 (16.0)	333 (22.8)	158 (9.8)		
	Body mass index: mean (SD)	28.7 (4.8)	28.9 (4.3)	28.5 (5.2)	0.02	0.1
Enabling resources	Income^b^: mean (SD)	4,299.1 (2,053.9)	4,483.4 (2,070.9)	4,123.5 (2,022.7)	<0.01	13.1
	Health insurance: n (%)					
	Statutory	2,859 (92.2)	1,323 (90.0)	1,536 (94.2)	<0.01	0.8
	Private	241 (7.8)	147 (10.0)	94 (5.8)		
	Lubben social network scale: mean (SD)					
	Family	9.4 (2.9)	9.3 (2.9)	9.4 (2.9)	0.31	0.5
	Friends	7.6 (3.4)	7.5 (3.5)	7.7 (3.3)	0.08	0.7
Need	CIRS-G score: mean (SD)	6.9 (5.4)	7.1 (5.6)	6.6 (5.3)	0.02	16.8
	SF-12 summary scores: mean (SD)					
	Mental component summary (MCS) score	47.8 (9.8)	49.0 (9.0)	46.7 (10.3)	<0.01	3.3

^a^Differences in proportions: *χ*^2^-test; differences in means: *t*-test; ^b^square root equivalent scale for 3 months (SRES).

Missing values in items of the health economic questionnaire and dosage of medication were not part of the multiple imputation model since these variables were too numerous and too varied to be imputed meaningfully. Therefore costs of medication with missing values for dosage were calculated using a conservative rule, whereby the pharmacy retail price of one package of the drug per 3 months was applied. Missing values in the resource use questionnaire were set at zeros, resulting in a conservative estimation of the health care service use and costs.

### Statistical analysis

“To assess the associations of covariates with health care costs, multiple Tobit regression models with marginal effects for the unconditional expected value E[y] of the dependent variable were estimated. Tobit regression models were used because resource use and cost data as used in our study often fall under the category of so-called corner solution outcomes. That is, the dependent variable “…y takes on the value zero with positive probability but is a continuous random variable over strictly positive values” [56]. In contrast to censored data where the “real” values for the zeros are unknown, data observability is no issue in corner solution applications. Following Woolridge’s [56] suggestions, we decided to present the marginal effects for the unconditional expected value E[y] of the dependent variable. These marginal effects are the most useful and informative measure since they provide information which is valid for the whole study sample. For the estimation of the pooled results, necessary because of the multiple imputed data, the software MIM was used [57]. Pseudo-R^2^s and Chi^2^s were estimated with reference to [58]. The level of significance was set at α = 0.05. All statistical analyses were performed using STATA Release 12 [52].

## Results

### Sociodemographic characteristics and missing values

The sample consisted of 3,124 respondents of whom 1,481 (47.4%) were male. The mean age was 69.6 years. Most of the respondents were married (71.8%), had less than 10 years of schooling (66.2%) and were covered by statutory health insurance (92.2%). The mean BMI was 28.7 and the mean SRES income for 3 months was 4,299 €. The mean CIRS-G score was 6.9 and the mean SF-12 MCS score was 47.8. Compared to women, men were slightly older, more often married, had more years of schooling, had a slightly higher BMI, had a higher income, were privately insured more frequently and had a higher level of multimorbidity (CIRS-G score) as well as a slightly better mental health status (SF-12 MCS score) (all p < 0.05).

Rates of missing values were highest on the income variable (13%) and the CIRS-G (17%). On all other variables missing rates were rather negligible with 0% for age and 3.3% for the SF-12 MCS score. Nevertheless, the missing values on all variables cumulated to a number of 681 (21.8%) incomplete cases. For further details of socio-demographic and health-related sample characteristics see Table 2.

### Health care utilization

98% of the respondents consumed at least one health care service or good during the 3 months preceding the interview (Table 3). The highest rates of service utilization appeared for outpatient physician services (95%) and pharmaceuticals (85%). Inpatient care was used by 9%. Non-physician providers, medical supplies and dental prostheses were used by 20% and 24%, respectively. The utilization of nursing care was rather low with 0.9% for formal and 1.7% for informal care. The utilization rates for services and goods tended to be slightly higher for women.

**Table 3:** Service utilization and costs in € (year 2009 values) during 3-month period

	**Number (%) of users**			**Mean costs in € per respondent (SD)**			
**Resource**	**All**	**Male**	**Female**	**All**	**Male**	**Female**	**p-value**^**a**^
	**n = 3,124**	**n = 1,481**	**n = 1,643**	**n = 3,124**	**n = 1,481**	**n = 1,643**	
Inpatient care	272 (8.7)	129 (8.7)	143 (8.7)	393.3 (1850.3)	379.4 (1773.2)	405.9 (1917.6)	0.69
Outpatient care							
Outpatient physician services	2,973 (95.2)	1,403 (94.7)	1,570 (95.6)	174.2 (234.7)	169.2 (261.9)	178.7 (207.1)	0.26
Non-physician providers	633 (20.3)	255 (17.2)	378 (23.0)	29.3 (92.6)	24.6 (70.2)	33.5 (108.7)	0.01
Medical supplies and dental prostheses	750 (24.0)	315 (21.3)	435 (26.5)	68.6 (412.7)	65.1 (442.8)	71.7 (383.5)	0.66
Pharmaceuticals	2,688 (85.4)	1,262 (85.2)	1,406 (85.6)	171.1 (290.7)	182.9 (303.2)	160.5 (278.6)	0.03
Nursing care							
Formal	29 (0.9)	10 (0.7)	19 (1.2)	8.44 (155.7)	6.8 (174.8)	9.9 (136.2)	0.59
Informal	54 (1.7)	23 (1.6)	31 (1.9)	43.5 (547.1)	62.5 (715.6)	26.4 (327.1)	0.07
Any service or good	3,061 (98.0)	1,445 (97.6)	1,616 (98.4)	888.5 (2181.6)	890.5 (2225.0)	886.6 (2142.4)	0.96

^a^Simple OLS regression with bootstrapped standard errors (4,000 replications).

### Health care costs

The mean total costs per respondent for the 3-month period were 889 € (Table 3). With 393 € per respondent, inpatient care was the sector with the highest mean costs followed by outpatient physician services (174 €) and pharmaceuticals (171 €). Mean costs of medical supplies and dental prostheses (69 €), informal nursing care (44 €) and non-physician providers (29 €) were comparatively low. With 8 € per respondent, mean costs of formal nursing care were lowest. Mean costs of non-physician providers were significantly higher for females (72 € vs. 65 €), while mean costs of pharmaceuticals were significantly higher for males (183 € vs. 161 €).

### Health care costs of users

Looking at mean health care costs for the 3-month period of only those respondents who used respectively services or goods, inpatient care (4,518 €), followed by informal nursing care (2,515 €) and formal nursing care (909 €), were the three most costly sectors (Table 4). Mean costs per user in all other sectors were comparatively low with 286 € for medical supplies and dental prostheses, 200 € for pharmaceuticals, 183 € for outpatient physician service and 145 € for non-physician providers. For pharmaceuticals and informal nursing care, the mean costs for users of health care services were significantly higher in males than in females.

**Table 4:** Costs in € (year 2009 values) during 3-month period for users of respective services

	**Mean costs in € per respondent (SD)**			
**Resource**	**All**	**Male**	**Female**	**p-value**^**a**^
	**n = varies**	**n = varies**	**n = varies**	
Inpatient care	4,517.7 (4,555.4)	4,355.6 (4,347.4)	4,663 (4,745.7)	0.56
Outpatient care				
Outpatient physician services	183.0 (237.2)	178.6 (266.0)	187.0 (208.2)	0.35
Non-physician providers	144.6 (160.1)	143.1 (108.3)	146.1 (181.4)	0.83
Medical supplies and dental prostheses	285.6 (804.9)	306.1 (922.1)	270.8 (708.8)	0.57
Pharmaceuticals	200.4 (305.1)	214.7 (318.0)	187.6 (292.6)	0.02
Nursing care				
Formal	909.2 (1361.9)	1,011.0 (1,974.1)	855.7 (963.3)	0.81
Informal	2,515.8 (3,361.4)	4,021.5 (4,219.4)	1,398.6 (1,968.5)	<0.01
Any service or good	906.8 (2,200.2)	912.7 (2,248.1)	901.4 (2,157.1)	0.89

^a^Simple OLS regression with bootstrapped standard errors (4,000 replications).

### Regression analyses

The results of the regression analyses (Table 5) revealed that the CIRS-G score and the SF-12 MCS score, representing the need factor in the Andersen model, were consistently associated with total, inpatient, outpatient and nursing costs: A one point increase in the CIRS-G score was associated with an increase of 18 € in 3-month inpatient costs, 17 € in outpatient costs and 3 € in nursing costs. This aggregates to an increase of the total costs of 41 € per score point of the CIRS-G. The SF-12 MCS score was inversely related to all cost sectors, resulting in a total cost decrease of 14 € per MCS score point.

Of the socio-demographic variables representing predisposing factors in the Andersen model, only respondents’ age was significantly positively associated with inpatient costs (10 € per year of age), outpatient costs (3 € per year of age), nursing costs (3 €) and total costs (13 €). The BMI was associated significantly with outpatient costs, with a 6 € increase per kg/m^2^ and total costs with a 11 € increase per kg/m^2^. 12 or more years of schooling significantly decreased inpatient costs by an amount of 161 €. Neither income nor health insurance status or Lubbens social network scale, representing enabling factors of the Andersen model, were associated with costs in any sector.

Denoted by the constants of the four regression models, the average 3-month cost per respondent, controlled for all covariates, was 843 € for total costs; 349 € for inpatient costs; 447 € for outpatient costs and 47 € for nursing costs.

**Table 5:** Multiple Tobit regression analyses with mean costs in 3-month period in € (year 2008 values) as dependent variable for total costs and by health care sector

	**Predictor variables**	**Total costs**			**Inpatient costs**			**Outpatient costs**			**Nursing costs**		
		**b (SE) (p-value)**^**a**^			**b (SE) (p-value)**^**a**^			**b (SE) (p-value)**^**a**^			**b (SE) (p-value)**^**a**^		
Predisposing factors	Age (centered)	13.33**	**(4.33)**	**(0.002)**	9.51*	**(4.34)**	**(0.028)**	3.44*	**(1.41)**	**(0.015)**	2.70***	**(0.62)**	**(0.000)**
	Female gender (ref: male)	21.65	(55.06)	(0.694)	53.46	(55.52)	(0.336)	-12.91	(17.89)	(0.471)	-2.70	(6.58)	(0.682)
	Marital status (ref: married)												
	Single	139.92	(148.79)	(0.347)	208.98	(187.53)	(0.265)	-5.57	(45.92)	(0.903)	30.72	(30.40)	(0.312)
	Divorced	103.85	(104.36)	(0.320)	246.97	(139.17)	(0.076)	-23.71	(32.22)	(0.462)	6.68	(15.69)	(0.670)
	Widowed	79.49	(74.99)	(0.289)	146.20	(84.96)	(0.085)	-16.02	(23.70)	(0.499)	6.57	(8.98)	(0.465)
	Education (ref: ≤ 9 years of schooling)												
	10-11 years of schooling	-14.95	(69.77)	(0.830)	-46.73	(65.42)	(0.475)	41.26	(23.29)	(0.077)	9.80	(10.64)	(0.357)
	≥ 12 years of schooling	-122.42	(76.53)	(0.110)	-160.88**	**(62.54)**	**(0.010)**	-26.87	(25.35)	(0.289)	2.40	(10.25)	(0.815)
	Body mass index^c^	11.37*	**(5.37)**	**(0.034)**	1.74	(5.25)	(0.740)	5.88**	**(1.75)**	**(0.001)**	0.54	(0.59)	(0.362)
Enabling	Income^b c^	0.02	(0.043)	(0.653)	0.03	(0.04)	(0.505)	0.02	(0.014)	(0.107)	-0.01	(0.01)	(0.382)
	Private health insurance (ref: statutory)	146.31	(106.51)	(0.170)	175.79	(130.97)	(0.180)	54.08	(34.31)	(0.115)	0.85	(14.65)	(0.954)
	Lubben social network scale^c^	4.53	(5.17)	(0.381)	5.92	(5.06)	(0.242)	-1.01	(1.68)	(0.548)	0.31	(0.58)	(0.596)
Need	CIRS-G score^c^	41.18***	**(5.05)**	**(0.000)**	18.23***	**(4.77)**	**(0.000)**	17.19***	**(1.64)**	**(0.000)**	3.05***	**(0.68)**	**(0.000)**
	SF-12 Mental component summary (MCS)^c^	-13.90***	**(2.72)**	**(0.000)**	-8.71***	**(2.59)**	**(0.001)**	-3.56***	**(0.89)**	**(0.000)**	-1.14***	**(0.33)**	**(0.001)**
Averaged pseudo R^2^		0.003			0.008			0.004			0.007		
Averaged Chi^2^		158.04			58.52			195.52			139.65		

^a^Multiple Tobit regression with marginal effects for the unconditional expected value E[y] of the dependent variable; ^b^square root equivalence scale for 3 months; *p <0 .05; **p < 0.01; ***p < 0.001; SE = standard error; ^c^the variable was mean centered; N = 3,124 with multiple imputation, imputation steps 100, cycle length 200. The bold data denotes those effects being significant.

## Discussion

In our study we analyzed health care costs of the elderly population in Germany. In order to organize and categorize the multiple factors which may influence health care utilization, we applied the theoretical framework developed by Andersen and Newman which distinguishes predisposing, enabling and need factors.

The main finding of our study is that the need factor of the Andersen model, operationalized through a measure of MM (CIRS-G) and complemented by a measure of mental health status (SF-12 MSC), was the dominant and most consistent predictor of health care costs. In our study a one point increase of the CIRS-G was associated with an increase in total costs of 41 € per 3 months. The finding that higher levels of MM lead to higher health care costs is in line with most comparable studies conducted in elderly populations and emphasizes the relevance of elderly’s need of health care. In a systematic review of the international literature, Lehnert et al. [20] found ample evidence of a positive association between MM and health care costs. Similar to our study, hospital stays [18,59], physician visits [60,61] and pharmaceuticals [62-64] were reported to elevate health care costs with each additional chronic condition.

Over and above the need factor, predisposing and enabling factors explained only little of the variance of costs. Of the predisposing factors, only age, the BMI and 12 or more years of schooling were found to be significant predictors of costs. The association found between age and costs seems to contradict the finding of a review conducted by de Boer [25], which found no definite influence of age on health care utilization in her review. However, de Boer’s findings apply to populations of chronically ill people, whereas our findings are based on a representative random sample of the elderly German general population. Furthermore, in a recent review the direction of the association between age and the utilization of health care services was found to be dependent on specific characteristics of the study population [21]. Of all other variables characterized as predisposing factors in the Andersen model, only the BMI demonstrated a cost-increasing effect of potentially inadequate health behavior. Yet, this only holds for outpatient and total costs. We could not detect a significant cost-increasing effect of female gender [22,65] or high education [66,67] as reported for health service use by other studies that applied the Andersen model. Instead, we found a cost-reducing effect of 12 or more years of schooling on inpatient costs with an amount of 161 €. Nor did we find a significant association of marital status and health care costs; yet, associations of marital status and health service use have been reported in different directions by other studies applying the Andersen model [68,69].

Neither respondents’ income nor their health insurance status, representing enabling factors of the Andersen model, was significantly associated with health care costs. By contrast, studies conducted in other countries, in particular the USA [66,70,71] , have repeatedly reported positive associations of insurance, income and costs. Since in our study the difference between statutory and private health insurance regarding total costs was rather high (227 €), the lack of significance of this effect might be caused by the relatively small number of privately insured respondents (7.8%). Yet, the lack of association of income and costs may be due to the specifics of the German health care system which is characterized by relatively low financial barriers to the utilization of comprehensive health care services (in particular by limiting co-payments to a maximum of 2% of income), despite undoubtedly existent income inequalities.

Possible reasons why predisposing and enabling factors explained only little of the variance in costs may be insufficient operationalization and variable selection for the predisposing and enabling factors. While there is no fixed set of predisposing and enabling variables defined for the Andersen model, our choice of variables was strongly oriented by Andersen’s suggestions, thereby trying to keep the regression models preferably parsimonious. However, as pointed out by Babitsch [21], an unambiguous assignment of a variable to one single factor is not always possible. In fact, we used variables similar to those used by other studies that applied Andersen’s framework: a recent review of studies that applied the Andersen model found the most frequently used variables for the predisposing factor to be age, marital status, gender, education and ethnicity [21]. We included all of them except for ethnicity, which might not be as relevant in Germany as, for example, in. the USA [66,70], but still may have explained some additional variance in costs. For the predisposing factor, the most frequently used variables reported by the mentioned review were income, health insurance and having a usual family doctor. We did not include the latter variable, as the study sample was recruited via their GPs. Yet we followed the suggestion to expand Andersen’s original model by the inclusion of the social network, following Pescosolido’s Network-Episode Model [72] “larger, more supportive networks decrease the use of patterns of care”. Although not significant, the effect of the Lubben Social Network Scale characterizes the Social Network as a potential cost driver in our study with 5 € per scale point. This might be due to the fact that compared to network analysis as practiced by Pescosolido, the Lubben scale is a rather basic construct and therefore potentially unable to depict the expected associations. Furthermore, the impact of the Social Network may depend on cultural aspects.

Mean total costs per respondent for a 3-month period were 889 € which - extrapolated to a 12 month period – corresponds to 3556 € per year. This figure is similar to annual total costs of 3315 € and 3730 €, respectively, recently reported by Nagl et al. [73] and Heinrich et al. [74] for similar samples of the elderly population in Germany. Similar to a study conducted in Germany by Nagl et al. [73], we found inpatient care, outpatient care and pharmaceuticals to be the three most costly health care sectors. However, amounting to 25% in the study by Nagl et al., their proportion of inpatient care was 19% lower than in our study. This might have been caused by the different time period of 12 months used by Nagl et al. vs. the 3-month period in our study. Since the utilization of inpatient care is a much rarer event than the use of outpatient care and pharmaceuticals, this in conjunction with the implementation of face to face interviews by trained study physicians in our study could have led to a reduction of recall bias and therefore more valid representation of inpatient costs as opposed to the study by Nagl et al., which used telephone interviews.

### Limitations

Our study is based on a large and nearly representative sample of the German elderly population [23,24]. Yet the number of users of nursing care in our sample was very low. This is likely due to the inability or unwillingness of nursing care recipients to complete a 2-hour home interview. A consequence of this selection process would be the underrepresentation of those with the highest level of MM and therefore an underestimation of the health care costs. This might have also influenced the prediction of the health care costs in the regression analysis.

We collected comprehensive data on health service utilization from a societal perspective. Yet the period of 3 months for which data was collected was rather short, possibly increasing the variance of calculated health care costs, which may be a further reason for the variance explained by the models being only small. On the other hand, this short period likely may have enhanced the accuracy of collected information about health care utilization because of less memory bias in participants’ responses.

We used detailed information on morbidity from which we calculated a well-established and - due to the assessment by GPs - objective measure of MM. How to best measure MM in this context is uncertain and needs further investigation. Research comparing the predictive ability of various MM measures on different health care related outcomes has produced inconclusive results [75-79], suggesting that no single measure of MM will completely capture the differences in the study subjects’ underlying illness level.

The conclusions from the results of the regression models hold only under the assumption that all missing values were at random (MAR). The cross-sectional study design provides only limited evidence of the causal associations between predictor and outcome variables.

## Conclusions

MM and mental health status, both representing the need factor in the Andersen model, were the dominant predictors of health care costs. Predisposing and enabling factors had comparatively little impact on health care cost, possibly due to specific conditions of the German health care system. Overall, the variables used in the Andersen model explained only a small proportion of the total variance in health care costs. Whether this indicates room for further improvement of the Andersen model or its limited ability to predict costs in general should be investigated in future studies. Different combinations of potential predictors of costs should be tested in various health care systems, as service use and costs in Germany are likely to differ from other countries. Nevertheless, in view of the predicted demographic change in developed countries, MM as a major cost driver has to be considered a key factor when planning future resource allocation. The development and implementation of integrated care models for patients with multiple chronic diseases and preventive programs aiming at modifiable risk factors could be options to reduce the financial strain of multimorbidity on health care systems.

## Competing interests

The authors declare that they have no competing interests.

## Authors’ contributions

DH contributed to the design of the study, analysis and interpretation of data, manuscript draft, and revision. HMa contributed to the analysis and interpretation of data, and manuscript and revision. HMü and KUS coordinated data collection, and the acquisition of data. RQ, WEH and BW contributed to the design of the study, and manuscript revision. TL contributed to manuscript revision. HB led on overall design of the study, and contributed to the manuscript revision. HHK contributed to the design of the study, interpretation of data, manuscript draft, and revision. All authors have approved the final manuscript.

## Pre-publication history

The pre-publication history for this paper can be accessed here:

http://www.biomedcentral.com/1472-6963/14/71/prepub
